# A New Method of Treating the Vegetable Parasitic Diseases of the Skin

**Published:** 1888-01

**Authors:** H. J. Reynolds

**Affiliations:** Professor of Dermatology in the College of Physicians and Surgeons; 163 State Street


The Medical Journal and Examiner.

VOLUME LVI. JANUARY, 1888. NUMBER 1.

### A NEW METHOD OF TREATING THE VEGETABLE PARASITIC DISEASES OF THE SKIN.

BY H. J. REYNOLDS, M. D.,

PROFESSOR OF DERMATOLOGY IN THE COLLEGE OF PHYSICIANS AND SURGEONS.

[Read before the Section of Dermatology, Ninth International Medical Congress, Washington.]

In this paper I propose to consider the treatment of what are commonly known among dermatologists as the vegetable parasitic diseases, viz., tinea favosa, tinea versicolor, and the three forms of tinea trichophytina. As the form of tinea trichophytina known as tinea circinata, or ordinary ringworm, and tinea versicolor are very superficial, they are very readily cured by any of the ordinary methods. The method I am about to describe will be more applicable to those wherein the deeper structures, as the hair follicles, etc., are involved, viz., favus (ringworm of the scalp), and barber's itch.

In these latter diseases many different methods of treatment have been suggested and tried from time to time, but they are usually very unyielding to treatment, and particularly so unless all the diseased hairs be systematically removed. For this reason I feel warranted in assuming that a suggestion regarding a method which to me bids fair to be preferable to any other, and which has never before, to my knowledge, been referred to, will be tolerated, as also a brief report of the few cases which I have treated in this manner.

The great object in all methods is to apply the parasiticide in such a manner as to insure its penetration to the bottom of the hair follicles and into the hair structure itself beneath the surface of the skin. Theoretically, and I think practically, likewise, the method I am about to describe, accomplishes this purpose.

There is a long-recognized law in Electro-Physics, in accordance with which fluids in a galvanic current move from the positive to the negative pole. It was demonstrated years ago, and repeatedly, that the absorption of medicinal substances may be induced by applying this principle, viz., by applying the substance to be absorbed on the positive electrode, placing it on the skin, and completing the circuit by placing the negative electrode on some other part of the body. This is, perhaps, best substantiated by the fact that a solution of cocaine applied to the surface of the sound integument in this manner, will, in a very few minutes, so permeate the skin as to produce complete local anæsthesia of all the cutaneous and subcutaneous structures covered by the positive electrode. On this subject I read a paper at the last meeting of the American Medical Association (Journal of American Medical Association, August 20, 1887). It was, in fact, in making these local anæsthesia experiments by this method, that the thought first occurred to me that the same principle might be used to advantage in the treatment of these very stubborn and annoying maladies.

The means employed in the method which I am about to describe to induce the penetration of the parasiticide are the galvanic current; though by this method it is possible also that the electricity itself has a salutary effect upon the diseased condition, or, in other words, has a destructive influence upon the fungus giving rise to the disease. With these prefatory remarks, I shall now proceed to describe the method as I have employed it.

Battery, Strength of Current, etc. —Though not an expert electrician myself, I believe it is an established fact that a large number of small cells gives more electrolytic action than the same area of large cells; I have, therefore, used a battery constructed on this principle—the same instrument, in fact, which I use for the removal of superfluous hairs by electrolysis — known as the McIntosh battery. For reasons not necessary to discuss at present, the Faradic current will not answer, neither will the negative pole of the galvanic. The strength of the current must vary with the sensitiveness of the part, the size of the electrode, etc. It is always best to use the strongest current that can be borne without discomfort to the patient, which, with the battery I use, when in good order, varies from five to ten cells. No universal rule cân be laid down, the safest guide being the feelings of the patient and the experience and discretion of the physician. For instance, to state a given number of cells would be inaccurate, inasmuch as the strength of the cell always varies with the freshness of the battery fluid, the length of time the plates have been in use, etc. To specify it as so many milliamperes would be unscientific, without giving the size of the electrode or stating the amount of the resistance. When there is much irritation or hyperæmia a very strong current will not be tolerated. It is not necessary that it should, as there is then less resistance. The sponge on the electrode used for the parasiticide solution should be of the finest and softest quality.

Mode of Application.—The surface to be treated should first be thoroughly cleansed of crusts, scales and sebaceous matter, by the usual process of oiling and washing with soap and hot water, etc. If thought necessary, of course, the loose hairs may be removed, though in the cases I have treated this was not done. I saturate the sponge of the positive electrode with whatever parasiticide lotion is preferred, which may be aqueous, alcoholic or ethereal, and place it directly upon the part to be treated. I then place the negative electrode, well saturated with water, on some point near by. A more remote point, as the hand, for instance, will answer, but it will take longer time to get the same effect than when placed near by. The electrodes should be kept firmly pressed to the skin, the positive being occasionally moistened, as required, with more of the solution and the negative with more water. In order to get a sufficient effect, the electrode should remain on each place several minutes before applying it to another place on the scalp. I think it is not wise to continue the treatment over ten or fifteen minutes in all at each sitting, and perhaps not oftener than once a day. The parasiticide used in the cases I treated was a one per centum solution of bichloride of mercury.

I had hoped to be able to report a considerable number of cases, but I have, as yet, only been able to treat three patients by this method—one of favus and two of tinea tonsurans.

CASE I. John A., æt. seventeen years, otherwise in fair health, was first seen January 12, 1887. He had had eruption on his scalp for ten years, during which time he had been treated by numerous physicians without receiving any permanent benefit. I ordered all local treatment and washing of the scalp to be suspended for two weeks. January 26, examination revealed a patch midway between vertex and forehead, about two inches in diameter, covered more or less with the characteristic yellow sulphur-colored crusts, showing in many places distinctly the cup-shaped character. The hairs were dry, brittle, broken off and loose, and a considerable amount of hair had been lost. There were also two or three other small patches similar in character. Examination with the microscope showed the crusts to be composed of the characteristic spores and mycilia.

The part was thoroughly cleansed and the treatment begun as described and continued daily. In addition, an ointment of one part of citrine ointment to four of lard was applied every night, and in the following morning the scalp was thoroughly cleansed preparatory to the electrical treatment. This treatment was carried out daily until February 1. The patient was not again seen until the latter part of May, when he was found to be entirely free from the disease.

CASE II. Mary L., æt. ten years, applied for treatment February 21, 1887. She had scaly patches on her scalp for four years — one patch in the region of the parietal eminence of right side about two inches in diameter, and two other smaller patches. The hairs were broken off, etc. The microscope revealed the trichophyton in and around the hairs. Treatment was the same as in Case I, except that no ointment was applied, and was continued almost daily for three weeks. June 12, the patient has had no treatment since March 14 and has been well ever since.

CASE III. Susan L., æt. twelve years, sister to Case II., applied for treatment at the same time. She had had a disease similar to her sister's, though not to quite the same extent, for two years and a half. Diagnosis was also verified with the microscope. Treatment was commenced at same time as her sister, and continued in the same manner for the same length of time. The result was complete recovery.

Although I have been unable, from the very limited number of cases treated, to determine to my entire satisfaction the full merits of the method, or the comparative merits of this and other methods, I am, nevertheless, satisfied that I accomplished results in these cases that I could not have attained by any other line of treatment such as I was before accustomed to try, and I therefore feel warranted in recommending its adoption by those in dermatological practice, and I feel convinced that, with further experience, more extended observation and more general adoption of the method, it will be found superior to all others that have been tried.

163 STATE STREET.

